# SCNP identifies CLL donor subgroups by profiling functional immune signaling in the context of immune modulatory receptor expression

**DOI:** 10.1186/2051-1426-3-S2-P189

**Published:** 2015-11-04

**Authors:** Erwan Le Scolan, Christie Fanton, Andy Conroy, Albert Tai, Nguyen Tan, Brent Louie, Erik Evensen, Rachael Hawtin

**Affiliations:** 1 Nodality, South San Francisco, CA, USA

## Background

Antibody therapeutics targeting the Immune Modulatory Receptor (IMR) PD-1 have efficacy in multiple indications, and molecules targeting other IMRs are in development. Increased understanding of IMR biology is required to design rational combination therapies and identify biomarkers of response and toxicity. Here we applied Single Cell Network Profiling (SCNP) to assess functional signaling across immune cell subsets in the context of IMR expression, using PBMC of CLL and healthy donors (HD).

## Materials and methods

SCNP is a multiparametric flow cytometry-based technology enabling simultaneous analysis of signaling networks in primary human samples across immune cell subsets without cell subset isolation. CLL (n=20) and HD (n=10) PBMC were profiled to interrogate; a) expression patterns of multiple IMRs (PD-1, PD-L1, OX-40, 4-1BB, GITR, LAG-3, TIM3) across cell subsets including effector and central memory (EM, CM) T cells, b) cell subset specific signaling following modulation with IL-2, IL-10, IL-15, or TCR (anti-CD3/anti-CD28), and c) the effects of PI3Kd or BTK inhibitors. CLL and HD data were compared to identify dysfunctional IMR expression and signaling associated with disease.

## Results

IMR expression across HD was similar whereas expression was heterogeneous in CLL. PD-1 expression was elevated in CLL blasts and across all CLL T cell subsets including effector and naïve T cells. In contrast, PD-1 was expressed primarily in EM and CM T cells in HD samples. PD-L1 expression also was elevated in CLL blasts vs. HD B cells. Reduced TCR à p-ERK and p-Akt was observed in a CLL donor subgroup vs HD. Lower T cell signaling was not associated with increased PD-1 expression but trended with reduced TIM-3 expression. Contrasting with reduced TCR responsiveness, increased IL-2 àp-Stat5 was observed in CD8+ T cells in CLL. Cell signaling in the context of PD-1 expression identified functional differences in CLL. TCR signaling was uniformly reduced in HD PD-1+ vs PD-1-T cells, whereas this trend was not consistent in CLL. Inhibition of BTK resulted in specific reduction of TCRàp-S6 but not p-AKT response, whereas PI3Kd inhibition resulted in complete pathway coverage.

## Conclusion

These data highlight how applying SCNP to profile both IMR expression patterns and functional signaling across immune cell subsets can be applied to immuno-oncology drug development. Applications include interrogating disease mechanism, informing rational combination therapies and identifying patient subgroups that may benefit from these therapies.

